# Rubber Band Ligation: A New Treatment Option for External Hemorrhoids

**DOI:** 10.7759/cureus.89701

**Published:** 2025-08-09

**Authors:** Danielle Sadik, Jean-Felix St-Onge, Francis St-Onge, Mark Bertematti, Feras Othman, Eliyahu Shemesh

**Affiliations:** 1 Medicine, Burrell College of Osteopathic Medicine, Las Cruces, USA; 2 Medicine, St. George's University School of Medicine, St. George's, GRD; 3 Medicine, Northumbria University, Newcastle upon Tyne, GBR; 4 General Surgery, University of Miami, Coral Gables, USA; 5 Colorectal Surgery, Delray Medical Center, Delray Beach, USA

**Keywords:** conservative treatment for external hemorrhoids, external hemorrhoids treatment, hemorrhoid pain relief, hemorrhoid treatment under local anesthesia, minimally invasive procedure for hemorrhoids, non-surgical treatment for external hemorrhoids, rubber band ligation, rubber band ligation procedure, rubber band ligation under local anesthesia, hemorrhoids

## Abstract

Hemorrhoids are dilated veins in the anal canal, classified into internal and external types based on their location relative to the dentate line. External hemorrhoids originate inferior to the dentate line from the inferior hemorrhoidal plexus. They are covered by the anoderm and are highly innervated by somatic pain receptors, leading to significant pain, especially when thrombosed. The management of external hemorrhoids has been primarily conservative, including increased fiber intake, stool softeners, and topical corticosteroids. More invasive treatments like hemorrhoidectomy or rubber band ligation have been avoided due to the intense postoperative pain and complications associated with such procedures. This case report and small patient cohort suggest rubber band ligation can be used as a minimally invasive procedure for the treatment of external hemorrhoids. A patient presenting with symptomatic non-thrombosed external hemorrhoids underwent rubber band ligation after being educated on the risks and benefits of the procedure. Lidocaine was injected into the surrounding tissue to anesthetize the hemorrhoid prior to band ligation. The rubber band was applied to the hemorrhoid, cutting off its blood supply and leading to ischemia, necrosis, and eventual sloughing of the tissue. Based on the success experienced by the first patient, a small patient cohort was offered the same treatment. In this cohort of 50 patients, 50% reported no post-procedure discomfort, 44% had mild pain managed with over-the-counter (OTC) analgesics, and three patients experienced moderate-severe pain that resolved within a week. Overall, ~90% were satisfied with the technique and would opt for the procedure again. This study demonstrates that rubber band ligation combined with local anesthesia injection could be an alternative option to hemorrhoidectomy for individuals with symptomatic non-thrombosed external hemorrhoids.

## Introduction

Hemorrhoids are dilated veins in the anal canal and can be classified into two types: external and internal hemorrhoids. These are separated by the dentate line, which determines the epithelium of the overlying hemorrhoid and which nerves will receive the sensation [1]. 

Internal hemorrhoids are located proximal to the dentate line and arise from the middle and superior hemorrhoidal veins. They are covered by the columnar epithelium, which has visceral innervation and is typically painless. The typical presentation consists of painless rectal bleeding and prolapse. Internal hemorrhoids are further classified into four classes depending on the severity of their prolapse [2,3]: grade 1 (visualized on anoscopy; no prolapse below the dentate line), grade 2 (prolapse with defecation or straining; reduce spontaneously), grade 3 (prolapse with defecation or straining; require manual reduction), and grade 4 (irreducible and may strangulate). 

On the other hand, external hemorrhoids arise from the inferior hemorrhoidal plexus and are located distal to the dentate line. They are covered by the anoderm, which is modified squamous epithelium containing somatic pain receptors innervated by spinal nerves [1]. Due to this innervation, they are known to cause severe pain, especially if they thrombose, swell, bleed, or have an overlying skin ulceration [4]. Somatic pain receptors on external hemorrhoids make treatment quite painful. Therefore, management of external hemorrhoids has historically been more conservative, including increasing water and high fiber intake, stool softeners, and topical corticosteroid creams [5]. 

Symptomatic external hemorrhoids are characterized by pain, swelling, protruding tissue around the anus, itching, irritation, and bleeding [4]. Any individual can experience discomfort provoked by sitting, by wiping after bowel movements, or by the presence of excess tissue around the anus that makes hygiene more difficult and prolongs cleaning time. Historically, symptomatic external hemorrhoids that failed to respond to conservative treatments were considered candidates for surgical excision. In addition, band ligation of external hemorrhoids has generally been avoided due to concerns about severe post-procedure pain [3]. In our study, band ligation under local anesthesia provides an additional well-tolerated approach for the treatment of external hemorrhoids.

## Case presentation

A 65-year-old Caucasian male patient presented to the clinic with perianal swelling that was especially bothersome when wiping. He has a past medical history of osteoarthritis, hypertension, hyperlipidemia, and pre-diabetes. His surgical history is significant for a cholecystectomy in his 40s and a total knee replacement four years ago. He has a five-year history of hemorrhoids with a total of three exacerbations that were treated conservatively with stool softeners and a hydrocortisone hemorrhoid cream. The third exacerbation was refractory to conservative measures. After a week of conservative management, the hemorrhoid still caused discomfort while wiping after bowel movements and pruritus. Physical examination of the patient on the initial visit showed a right-sided lateral hemorrhoid partially obstructing the anal opening. There was no erythema or bleeding from the hemorrhoid, but it was tender when manipulated out of the way to perform a digital rectal examination (DRE). No internal hemorrhoids were identified during the DRE. The stool guaiac test was negative. Due to discomfort and hygienic concerns, the patient wished to have this mass removed. The patient expressed a preference for definitive management this time but declined surgical excision because he was worried about the healing time and potential infection risks. To address the patient's concerns about surgery, an improvised method involving local anesthesia followed by rubber band application to the affected area was developed.

Lidocaine in a small-gauge needle (27G) was injected into the surrounding tissue of the hemorrhoid. Once the area was completely numb, a rubber band was placed around the hemorrhoid, and blood flow was cut off to the tissue distally, leading to ischemia and subsequent necrosis.

After the procedure was completed, the patient was asked to stay in the office for 15 minutes to measure his comfort level. After the observation time was finished, he mentioned mild discomfort rated 4/10 but felt well enough to return home. The patient was instructed to also take over-the-counter pain medications and to come to the emergency department in the event of severe, intolerable pain. 

On their outpatient follow-up two weeks later, the area of concern was sloughed and left a small ulcer where the rubber band had fallen off. The patient claimed that he had mild discomfort ranging from 2/10 to 4/10 for about five days that required a sitz bath twice daily and Tylenol as needed, but by the fifth day, the pain was manageable with Tylenol as needed, and the rubber band fell off by the sixth day. He continued to use Tylenol for the remainder of the two weeks as needed for pain control and Neosporin ointment for the leftover ulcerated skin. It was then decided to apply this method to other patients who had similar presentations. 

**Figure 1:**
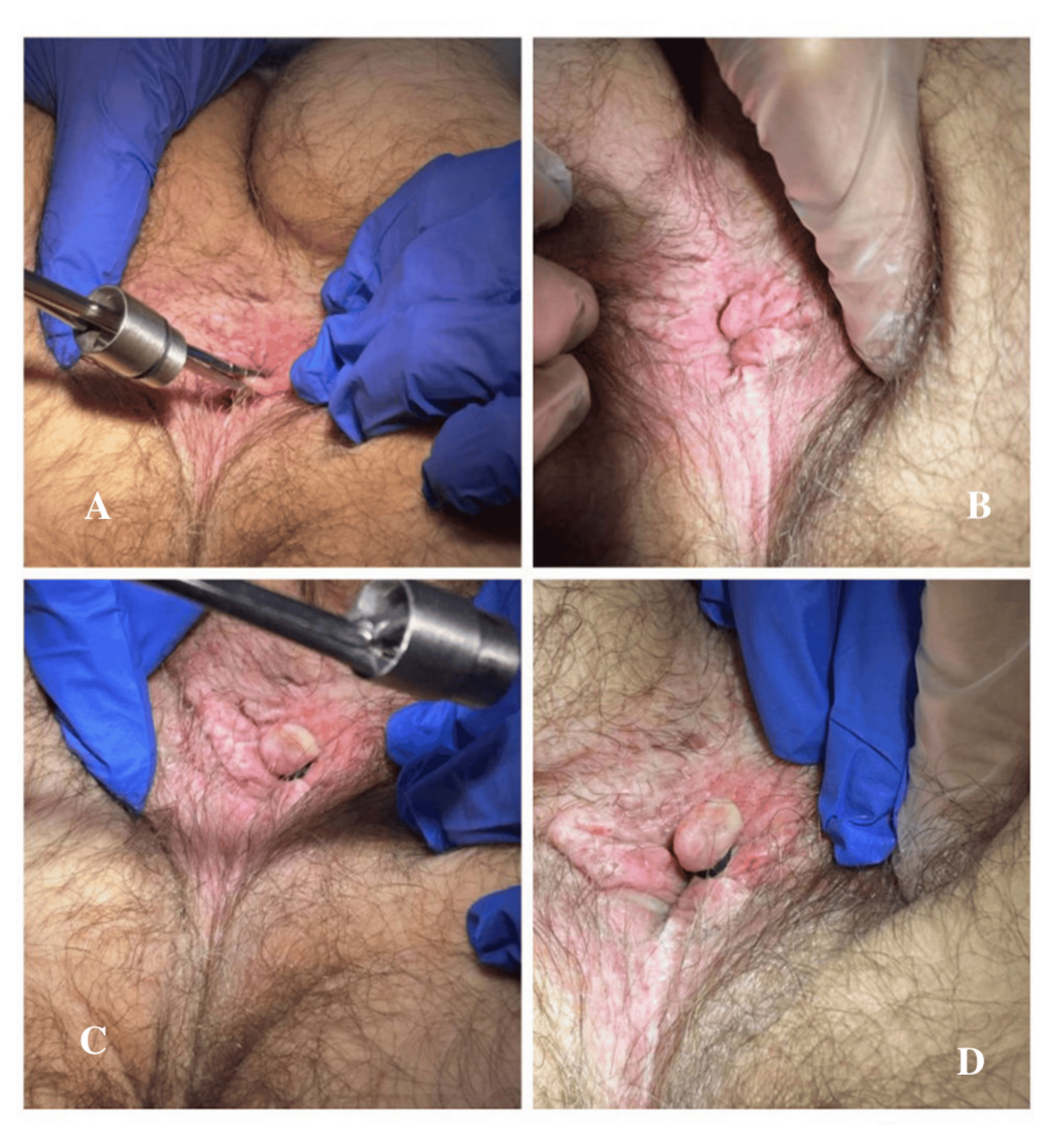
The procedure being done to a patient with a hemorrhoid on the lateral aspect. (A) The clamp with the rubber band holder placed onto the hemorrhoid. (B) Visualization of a hemorrhoid. (C and D) The secured rubber bands onto the hemorrhoid base. (D) Small puncture sites lateral to the hemorrhoid and the anus, where lidocaine was injected.

Due to the satisfaction of the patient, this procedure was repeated on a small cohort of 50 other patients with similar initial presentations. Similar outcomes as experienced by this patient were observed with this small cohort. In all 50 patients undergoing this procedure, two experienced premature rupture of the rubber band that needed to be debrided in-office, without further anesthesia. In addition, three patients had necrosed hemorrhoids that were associated with moderate-severe pain rated 5-7/10; this pain subsided during the first week (Figure 1).

## Discussion

External hemorrhoid management starts with conservative management, and depending on the severity of symptoms and of the hemorrhoid, nonoperative and operative approaches may be warranted. Acutely thrombosed external hemorrhoids and rapid stretching of the overlying skin during sudden distention can cause severe pain that may last for a few days [6]. Although our patient did not have an acutely thrombosed hemorrhoid, he experienced symptoms like itchiness, burning, and discomfort from cleaning after bowel movements. Furthermore, he was already advised on the various conservative measures possible, as this was his third episode. These measures involved diet and nutrition changes, stool softeners such as docusate, sitz baths, and pain control measures [5]. For most mild cases, increasing the diet with more fiber and fluid intake can help relieve constipation and reduce the recurrence of hemorrhoids [6]. Pain management for external hemorrhoids frequently involves topical agents, including over-the-counter creams and ointments containing hydrocortisone, phenylephrine, and pramoxine, which can provide symptomatic relief but should not be used independently [7]. While effective, conservative management may be associated with a longer duration of symptoms before the onset of relief and a high recurrence rate [2]. 

Unfortunately, nonoperative interventions such as sclerotherapy, cryotherapy, coagulation therapy using infrared photocoagulation, and rubber band ligation [1,8] are traditionally used for internal hemorrhoids only. This is because internal hemorrhoids originate above the dentate line, thereby having visceral innervation, and are painless. On the contrary, external hemorrhoids are located below the dentate line and contain somatic pain receptors, which makes them painful [1]. Therefore, nonoperative treatment as described can only be utilized on internal hemorrhoids. However, by injecting the external hemorrhoids with lidocaine prior to rubber band application, the somatic innervation and pain can be bypassed. Upon rubber band application, the somatic nerves in the hemorrhoid lose their blood supply and subsequently turn ischemic. During the first minutes to hours, the nerves will develop conduction block, neuropraxia, and paresthesia. If the rubber band is not removed after this stage, then the ischemic nerves will lose the ability to sense pain, fine touch, and temperature, rendering the hemorrhoids painless [5,9].

For patients who are presenting with acutely thrombosed external hemorrhoids within the first 2-3 days of symptom onset, surgical excision of the symptomatic hemorrhoid is often recommended [10]. This approach can provide rapid symptom relief, reduce recurrence rates, and extend remission intervals. However, some of the notable risks associated with hemorrhoidectomy are severe post-op pain and discomfort, especially with open procedures [8,11], persistent pain from nerve injury, infections, bruising, post-surgery urinary retention, and anal stenosis if there is excessive scarring. After the three-day window, most hemorrhoids begin to spontaneously resolve, and a hemorrhoidectomy becomes less beneficial due to the postoperative pain and complications that the patient may experience [10,12]. 

Our patient, and most of the patients who underwent rubber band ligation for external hemorrhoids, presented after the 2-3-day window of symptom onset. And, they did not have thrombosed hemorrhoids; thus, they were poor candidates for hemorrhoidectomy. Unfortunately, the patients were still complaining of discomfort while cleaning after a bowel movement, pruritus, and burning sensation. They were therefore offered rubber band ligation with local anesthetic. In our patient and cohort, similar benefits of pain resolution and decreased recurrence were seen without some of the surgical risks associated with hemorrhoidectomies [8,12].

The patient cohort consisted of 50 patients, of whom 25 had no perceived discomfort after the procedure and did not require additional pain management post-procedure. Twenty-two patients reported mild pain and discomfort, ranging from 1/10 to 4/10 pain scale. This was managed with ibuprofen or Tylenol PRN. Only three patients reported moderate-severe pain above 5/10. None of these three patients needed narcotic pain medication. Their pain improved daily and eventually regressed with similar clinical outcomes compared to the patient cohort. Two patients experienced premature rupture of the rubber band prior to day 5, which resulted in hemorrhoids that were not fully detached. They were both managed by in-office debridement of the remaining necrotic tissue with a scalpel. No anesthetics were required as the hemorrhoids were already necrosed and therefore not innervated with pain fibers anymore. After the debridement, no further complications and pain were noted in these individuals. No patients reported foreign body sensations. The pain was completely resolved within 5-7 days, after which the rubber band had fallen off for all patients within our cohort. Within 1-2 weeks, patients were asked to follow up for reassessment of the hemorrhoid and anus. A small superficial ulcer was noted at the location of the rubber band. No patient had any signs or symptoms of an infection, and no bleeding was noted in any patient. The patients in the cohort were satisfied with the procedure, and no recurrence was noted at any follow-up visit. About 90% returned for a second or third procedure on other hemorrhoids, if present, every 3-4 weeks.

Our case report and patient cohort demonstrate a promising new technique that may allow providers to use rubber band ligation for external hemorrhoids with good clinical outcomes. Nevertheless, the sample size of 50 individuals limits the external validity of this new technique to the public. In addition, our study did not extensively assess the conservative methods attempted, the duration they were attempted, the time of initial diagnosis, and the severity and recurrence of the hemorrhoids for each of the 50 patients in our cohort. Further investigations should be focused on analyzing the recurrence rate of hemorrhoids in patients who underwent rubber band ligation for external hemorrhoids. Although our patient cohort did not show any recurrence of hemorrhoids during the follow-up visits, this is a new method of managing patients with external hemorrhoids, and thus, the long-term recurrence remains unknown and undocumented.

## Conclusions

Historically, rubber band ligation has been limited to internal hemorrhoids only. However, our case study and small patient cohort have demonstrated that it can be used on external hemorrhoids with local anesthetics. The novel use of rubber band ligation for external hemorrhoids as a non-surgical procedure enables patients to avoid surgical excision and the many risks associated with such a procedure while simultaneously benefiting from similar clinical outcomes.

The procedure involved the application of lidocaine as a local nerve block to anesthetize the highly innervated perianal tissue prior to ligation. Our patient cohort of 50 individuals demonstrated that 50% of the patients experienced no discomfort post-procedure and the other 44% had mild pain that was controlled with ibuprofen or Tylenol. Three patients experienced moderate-severe pain, which gradually resolved within a week. Two cases had a premature rupture of the rubber band, which was debrided in-office and did not require any anesthetics. Approximately 90% individuals who received this treatment option were satisfied with this new technique, had no short-term recurrence, and stated they would undergo the procedure again for future hemorrhoids.

Conservative measures such as stool softeners, a high-fiber diet, and adequate hydration remain the first-line management for external hemorrhoids. However, rubber band ligation offers a more effective alternative to traditional nonoperative treatments, like topical agents, creams, or sitz baths, which provide only temporary relief and carry a high recurrence rate. On the other hand, surgical hemorrhoidectomy has a low recurrence rate but carries an important risk of postoperative pain and complications. Although limited by sample size and long-term recurrence rate, the use of rubber band ligation with local anesthesia in external hemorrhoids has shown promising findings in both clinical outcomes and, importantly, patient satisfaction.
